# Cost-effectiveness of a photopethysmographic procedure for screening for atrial fibrillation in 6 European countries

**DOI:** 10.1186/s13561-022-00362-2

**Published:** 2022-02-26

**Authors:** Steffen Wahler, Ralf Birkemeyer, Dimitrios Alexopoulos, Zbigniew Siudak, Alfred Müller, Johann-Matthias von der Schulenburg

**Affiliations:** 1 St. Bernward GmbH, Friedrich-Kirsten-Straße 40, D-22391 Hamburg, Germany; 2 Herzklinik Ulm, Magirusstraße 40, D-89077 Ulm, Germany; 3 Attikon University Hospital, National and Kapodistrian University of Athens Medical School, Tetrapoleos 18, GR-115 27 Athens, Greece; 4 Department of Internal Medicine and Cardiology, Jan Kochanowski University, Stefana Żeromskiego 5, PL-25-369 Kielce, Poland; 5 Analytic Services GmbH, Jahnstr. 34c, D-80469 Munich, Germany; 6 Universität Hannover, Welfengarten 1, D-30167 Hannover, Germany

**Keywords:** Atrial fibrillation, Screening, Prevention of stroke, Cost-effectiveness analysis

## Abstract

**Background:**

Strokes cause an estimated annual health care burden of 170 billion euros across Europe. Atrial fibrillation is one of the major risk factors for stroke and increases the individual risk 4.2-fold. But prevention with anticoagulants may reduce this risk by 70%. Screening methods are employed to detect previously undetected atrial fibrillation. Screening studies in various European countries show a high degree of undetected atrial fibrillation. This study aims to assess the cost-effectiveness of systematic screening with a smartphone application, named Preventicus Heartbeats. It is a hands-on screening tool for use on smartphone to diagnose AF with high sensitivity and specificity.

**Methods:**

A previously published model for calculating screening cost-effectiveness was extended to 6 European countries covering a wide range in terms of treatment costs and epidemiologic parameters.

**Results:**

The use of screening lowers the cost per case in countries with comparatively high levels of health care costs (Switzerland: -€75; UK: -€7). Moderate higher costs per case were observed in 4 countries (Greece: €6; Netherlands: €15). Low levels of health care costs result in less or no potential for further cost reduction (Poland: €20; Serbia: €33). In all countries considered, the model showed an increase in effectiveness measures both in the number of strokes avoided and the quality adjusted life years. The number of strokes avoided per 1000 participants ranged from 2.52 (Switzerland) to 4.44 (Poland). Quality-adjusted life-years per case gained from screening ranged from 0.0105 (Switzerland) to 0.0187 (Poland). The screening procedure dominated in two countries (Switzerland, UK). For the remaining countries, the incremental cost effectiveness ratio ranged from €489/QALY (Greece) to €2548/QALY (Serbia).

**Conclusion:**

The model results showed a strong dependence of the results on the country-specific costs for stroke treatment. The use of the investigated screening method is close to cost-neutral or cost-reducing in the Western European countries and Greece. In countries with low price levels, higher cost increases due to AF screening are to be expected. Lower costs of anticoagulation, which are expected due to the upcoming patent expiry of direct anticoagulants, have a positive effect on the cost result.

**Supplementary Information:**

The online version contains supplementary material available at 10.1186/s13561-022-00362-2.

## Introduction

International studies show a high-cost burden of stroke in Europe. A multinational study in 2017 estimated the direct cost burden of EUR 60 billion (=1.4% of total direct health care costs) [1]. The same study estimated the sum of direct and indirect costs at €169 billion. Earlier analyses [2] estimated the direct costs of stroke in the EU at a similar level, but [3] projected a 36% increase in the incidence of stroke between 2000 and 2025. This development is mainly due to the ageing of the population. In contrast, age-adjusted calculations of stroke incidence show a constant or decreasing trend [4]. Similar trends can be expected in the prevalence of atrial fibrillation due to age. Krijthe et.al [5]. estimate on the basis of Dutch results that the number of people with atrial fibrillation in the European Union (then 28 member states) will increase from 211 million in 2000 to 547 million in 2060.

Atrial fibrillation (AF) is the most common arrhythmia of clinical significance [6]. It is a supraventricular tachyarrhythmia with uncoordinated activity of the atria and frequencies between 350 to 600 bpm. Result is functional loss of activity of the atria with reduction of cardiac output [7]. AF is associated with increased morbidity, especially stroke and heart failure, and increased mortality [8–11] and constitutes a significant public health problem [12–14]. The prevalence of diagnosed AF is estimated 1% in Germany with increase in the old age (8% in population above 80 years) [15].

AF is mainly discovered in patients who seek medical treatment due to related clinical symptoms (palpitations, shortness of breath, etc.) or in previously asymptomatic patients after they have suffered a stroke which was possibly caused by cerebral embolism [6]. Due to the relatively short observation periods, e.g., only around 60 s with a usual resting ECG, some screening studies provide low detection rates of previously undetected AF.

AF/Stroke: AF is associated with higher mortality, and, if stroke occurs, AF patients suffer a significantly higher degree of disability, death and risk of a second stroke within 12 months compared to non-AF patients [16–20].

OAC/DOAC: Anticoagulating agents to reduce the risk of stroke with AF have been in clinical use since the 1980s. Several studies found oral anticoagulation to reduce the risk of stroke by 65–80% in patients with AF [21, 22]. Guidelines therefore require mandatory prevention with anticoagulants in AF patients with additional risk factors [23]. Vitamin K antagonists and antiplatelet agents have been increasingly replaced by direct (or “non-vitamin K antagonist”) oral anticoagulants (NOAC) in the last 5 yrs. They show a slightly improved effectiveness and a significantly improved safety profile compared with vitamin K antagonists, particularly with regard to bleeding [24–28].

Thus, systematically undetected AF is a systematic risk for stroke for patients who could otherwise benefit from an anticoagulation therapy. Therefore, early detection and appropriate measurements reduce the number and burden of strokes.

“Preventicus Heartbeats” is a Class 2a medical app with the purpose to detect and record the presence or absence of AF episodes by means of regular short measurements on the participant’s mobile phone. The technology is based on recordings of photopethysmographic (PPG) signals which is widely used for pulse detection. By simply putting a finger on the smartphone camera the pulse curve is recorded and automatically analyzed. Pathological reports are reviewed by a telecare center before indicating the result to the user. A training program on how to perform measurements is integrated in the app as well as aids and feedback tools. Sensitivity and specifity of atrial fibrillation detection compared to the gold standard electrocardiogram were determined in prospective validation studies [29, 30].. Participants diagnosed with absolute arrhythmia during the “AF screening” will undergo a validation phase of up to 2 wks. A continuously recording, telemetric chest ECG event recorder (“AF confirmation”) allows the final diagnosis and an appropriate treatment of AF according to the guidelines, by ruling out incorrect screening results or results that are not relevant for treatment, which may arise from short-term arrhythmia episodes during the mobile phone measurement.

There are strong differences between European countries in the epidemiology of atrial fibrillation and stroke [31–33]. European-wide cost studies (e.g. [1, 34, 35]) similarly show strong differences in health care cost levels and especially in the treatment of stroke. In the study presented here, a model framework developed for Germany and already published ([36]) was applied to 6 other European countries (UK, Netherlands, Switzerland, Greece, Poland, Serbia), which differ strongly in terms of both epidemiology and cost position.

## Methods

**Fig. 1:**
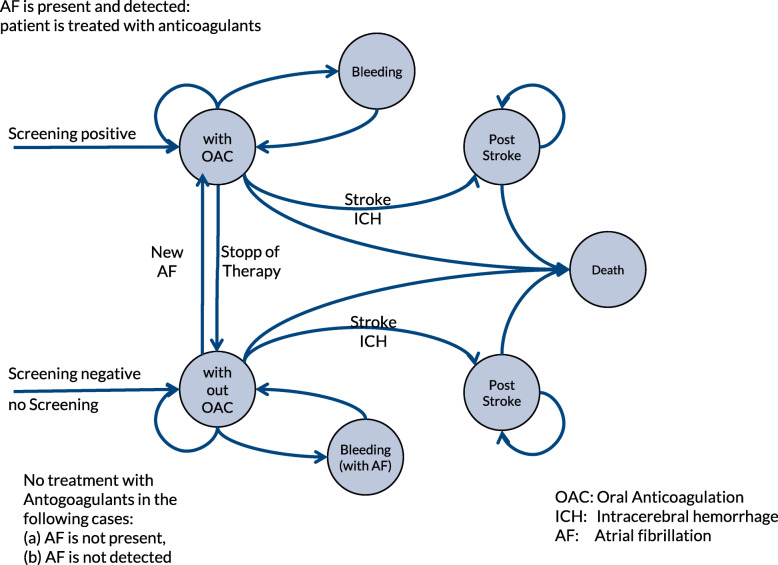
Markov model structure, adopted from [36]

To model the cost-effectiveness of using a screening method to prevent stroke, we adopted a published Markov cohort model [36] (see Fig. 1).

The model represented a hypothetical screening program among people insured by the statutory health insurance in Germany. In accordance with the ESC guidelines for the diagnosis of atrial fibrillation, this model was limited to the screening of insured persons aged 65 years and older [37].

In the treatment arm of the model, the target population is screened with Preventicus Heartbeats. Participants with abnormal heart rhythm patterns are then examined by ECG. After the ECG step identifies previously unrecognized AF, stroke prevention is performed with anticoagulation medication. Endpoints of the model are the number of strokes avoided and the effect of the procedure on quality of life measured by quality adjusted life years (QALY). The model considers gastrointestinal bleeding and increased likelihood of intracerebral hemorrhage as adverse events of anticoagulant treatment.

In the alternative branch of the model, patients with undetected AF have an increased incidence of stroke, which is associated with a lower quality-adjusted remaining lifetime. There is a cost tradeoff between the two branches: major cost components in the treatment branch are the screening costs and the costs for anticoagulation treatment. In the alternative branch, higher costs arise from the increased incidence of stroke.

Model endpoints are, on the cost side, the cost effect of the screening procedure from the perspective of the national payers. Effectiveness endpoints are the number of strokes avoided and the effect on quality of life. The model calculation is performed in the base case for 45 years after the screening time point, which generally corresponds to the remaining lifetime of people over 65 years of age. The sensitivity calculations deviate from this assumption.

The model parameters can be divided into three groups: effectiveness, costs and epidemiology. Regarding effectiveness (quality of the screening procedure, prevention of stroke, assessment of quality of life), the parameters of the model of [36] were adopted (Table 1). Screening and treatment costs and epidemiology require different assumptions for each of the countries considered.

**Table 1:** Efficacy parameters: Screening and OAC Efficacy *

Category	base case	Description
**Screening**		
Undetected AF in % detected AF	0.33	Assumption (prevention, 1/3 ratio undetected AF to detectedAF)
Sensitivity Preventicus screening	0.92	from DETECT AF [29]
Specificity Preventicus screening	0.99	from DETECT AF [29]
Positively validated screening results (after Holter ECG)	0.85	Adoption acc. to Wachter et.al [38].
**OAC Efficacy**		
Increased stroke rate with AF without prevention	4.20	mean: Wolf et.al [39]. SD: assumption
Reduction of stroke rate through prevention **	0.70	Assumptions, based on Hart [22] and López-López [24]

*Adopted from [36], Table 3

### Assumptions effectiveness

The Preventicus Heartbeats screening procedure has been found to have a sensitivity of 92% and a specificity of 99% (Detect AF). 85% of cases in which Preventicus Heartbeats detects AF are confirmed in the two-week validation phase (assumption according to [38]). Previous studies found a 60–85% reduction in stroke incidence in patients with drug-assisted stroke prevention compared with patients without stroke prevention [22, 24]. Table 1 contains a summary of the assumptions on effectiveness.

### Assumptions costs

The costs for Preventicus screening in Germany are €47.54 (as of June 2020) for the screening app and a further €279.50 per case for the 2-week validation of positive cases. These costs are also assumed for the Western European countries considered. Lower screening costs are assumed in the considered countries in Eastern and South-Eastern Europe (Poland, Serbia, Greece). The extent of price reduction is proportional to an OECD index of health care costs [35]. The basis for the costs of outpatient treatment in the preventive and acute phases are the costs of the statutory health insurance in Germany [41] according to the EBM catalogue. These costs were projected to the other countries considered using the OECD index mentioned above. The average prices for anticoagulants were researched for the countries considered (Supplement, Tables 1-2). For the DOAC share, 35% was assumed in the model following the results of [42].

The DRG catalogues of the countries under consideration were used to determine the inpatient stroke costs. In the DRG catalogues, the costs for the treatment of mild, moderate and severe cases were determined and weighted uniformly. Publications on hospital costs for stroke with divergent results were considered ([43–45]). The result was an index of inpatient stroke costs for the 6 countries considered (Supplement, Tables 3-4). Using this index, the cost assumptions for inpatient stroke costs from the model ([40], figures inflated by [36]) were projected to the other countries considered. Costs for rehabilitation, nursing and outpatient care after stroke were adjusted using the OECD healthcare price index [35] (see Table 2). Inflated case costs for Germany ([36, 40]) were split into inpatient and outpatient treatment. For the 6 countries considered, inpatient and outpatient costs were derived from the German data using the inpatient and outpatient cost indices for these countries (see Table 2).

**Table 2:** Cost parameters, by country

	Germany [36]	Switzerland	Greece	Netherlands	Poland	Serbia	UK (NHS England)
Outpatient costs (index, DE = 100) [OECD] [35]	100	180	88	126	44	43	123
Screening costs per app (screened patient)	47.54 €	47.54 €	*41.84 €	47.54 €	*20.92 €	*20.44 €	47.54 €
Validation cost	297.50 €	297.50 €	261.80 €	297.50 €	130.90 €	127.93 €	297.50 €
VKA (%) [42]	35%	35%	35%	35%	35%	35%	35%
OAC: Cost per day							
VKA cost/d	0.20 €	0.33 €	0.12 €	0.12 €	0.05 €	0.03 €	0.04 €
NOAC cost/d	3.00 €	3.39 €	2.49 €	3.43 €	2.76 €	3.51 €	2.68 €
Average inpatient stroke costs € per stay	4007 €	11,344 €	2864 €	5328 €	2050 €	803 €	4973 €
Inpatient costs/stroke (index, DE = 100)	100	283	71	133	49	20	124
Bleeding costs	Germany: 50 € (minor)/2025 € (major); country specific prices proportional to outpatient cost index						

Outpatient cost index: health price index, OECD 2014; levels normalized to German level (Germany = 100)

Screening costs: German costs (source Preventicus price list 06(2020) applied to Western European countries. For Greece, Serbia and Poland prices were adjusted proportionally to the OECD health price inces

OAC medication: government/health authority catalogues (Greece, Serbia, Netherlands, UK), retail prices (Poland, Switzerland, Germany); Inpatient cost index: rough estimate from DRG costs, stroke subsections (see Supplement, Tables 3 and 4)

*Adjusted to country-specific cost levels

In our previously published model for AF screening costs in Germany [36], cost returns from risk structure compensation were included in the cost-effectiveness analysis from the perspective of statutory health funds in Germany to reflect all financial flows related to the AF screening. However, the inclusion of risk structure compensation would lead to distortion in an international comparison and were therefore not considered in the current modelling. The model for Germany was recalculated without risk structure compensation effects, with otherwise unchanged model assumptions, to achieve consistency with the definition of end points in the study presented here.

### Assumptions side effects

The model considers bleeding events as side effects of treatment with oral anticoagulants: minor bleeding [46], major bleeding [27, 47] and intracranial bleeding [48]. The case cost of cerebral infarctions is assumed to be 1.5 times that of ischemic strokes. For Germany, severe bleeding is assigned the DRG cost of gastrointestinal bleeding (G-DRG catalogue numbers G70A-C and G46B [49]). For the other countries, these costs are extrapolated using the above-mentioned “inpatient index” (Table 2).

### Assumptions epidemiology

Eurostat data concerning the age structure show the differences in the countries under consideration. The proportion of people over 65 in the total population ranges from 17.5% (Poland) to 22% (Greece) (Table 3). Regarding gender-specific mortality, the differences between countries are even more pronounced. Assumptions on increased mortality after stroke are based on [50].

**Table 3:** Costs of stroke treatment per case, per year (model assumptions)

	Inpatient share of costs, year 1 [44]	Inpatient share of costs, subsequent years [40]	Stoke costs year 1	Stroke costs subsequent years
Germany [40], inflated [36]	36.2%	15.9%	21,060	6231
Switzerland			45,763	12,239
UK			25,980	7674
Netherlands			27,069	7921
Greece			17,236	5315
Poland			9648	2719
Serbia			7301	2451

Central epidemiological parameters of the model are the prevalence of atrial fibrillation and the incidence of stroke. For both parameters, community and health care studies are available in European countries with diverging results. In general, it is assumed that the incidence and prevalence rates will increase due to the aging of the population in Europe. In the case of stroke incidence, this effect overlays the opposite tendency of decreasing age-adjusted incidence rates due to preventive measures. Comparable estimates for epidemiological parameters were developed by the Global Burden of Disease (GBD) ([31]) study for the diagnoses of stroke and atrial fibrillation. However, Stroke incidence rate published by [31] differ from significantly from figures published for the 7 countries under consideration. GBD incidence rates for ischemic strokes in Germany are 35% lower compared to [51] and 30% lower compared to [52]. For stroke incidence, the model presented here uses the model results for total stroke reported by [51]. According to [41], the proportion of ischemic strokes in all strokes is 87%. Using the group frequencies published by [31], the incidences per country are broken down to individual groups (age in 5-year bands and sex). For the UK, these assumptions result in 117,000 ischemic strokes which matches the number of incidences estimated by [53]. Other comparisons to published results are listed in Table 4. Incidences of ischemic stroke per 100,000 used in the model vary from 176 (UK) to 493 (Serbia).

**Table 4:** Life expectancy, AF prevalence and stroke incidence in the overall population and in the simulated cohort

	Life expectancy years (simulated cohort)	AF prevalence (%) (total population)	AF prevalence (%) (simulated cohort)	Yield of screening (previously undetected AF) *	Stroke incidence (per 1000) (total population)	Stroke incidence (per 1000) (simulated cohort)
Switzerland	14.7	1.5%	6.7%	2.23%	2.01	7.0
UK	13.6	2.3%	10.2%	3.40%	1.76	5.5
Greece	13.7	2.2%	8.0%	2.67%	2.77	8.0
Netherlands	13.5	1.9%	8.1%	2.71%	1.78	5.5
Poland	12.9	2.2%	9.9%	3.30%	2.85	11.3
Serbia	10.8	1.7%	6.8%	2.27%	4.93	18.0
Germany	12.8	2.4%	9.1%	3.04%	2.71	8.9

*Expected share of previously undetected AF given the ratio 1/3 between the share of previously unknown vs. diagnosed AF

Simulated cohort: 65–85 years, determined by general age distribution and AF prevalence by age

The source for the AF prevalence rates are German sick fund data [54] which were applied to the other countries, with prevalence rates adjusted proportionally according to [31]. Table 4 summarizes the assumptions on epidemiology. The results for Germany (prevalence rate 2.4%) and UK (prevalence rate 2.3%) are slightly higher than the result of a multinational meta-analysis [55] (2.3 and 1.9%, respectively). A British government source [56] estimates the AF prevalence in England at 2.5%.

Little is known about the prevalence of undetected atrial fibrillation. Based on the results of [36, 57, 58] assumed a ratio of detected to undetected atrial fibrillation of 3:1. The model presented here adopted this ratio as the base case. The yield factor of the screening procedure (prevalence of unknown AF in the target population of at least 65-year-old) changes proportionally to the AF prevalence in the model and ranges from 2.2% (Switzerland, Serbia) to 3.4% (UK).

### Quality of life assumptions

For quality of life, the assumptions from [36] were adopted unchanged. Significant reductions in the quality of life are made in the case of first and repeated strokes as well as after cerebral hemorrhage. Further reductions take into account the age effect, the reduced quality of life in the presence of arrhythmia (reduced when taking oral anticoagulants) and the temporarily reduced quality of life in the presence of bleeding. The above assumptions apply globally to all countries considered.

### Other model assumptions

For each of the countries considered, separate model runs simulated the health development of cohorts with a given starting age of 65 to 85 years over the remaining lifetime (maximum 45 years). Cost and effectiveness results were discounted at 3%. The results for the sub-cohorts with different starting ages are applied to a notional total cohort whose age and sex distribution reflects the demographics of the countries considered and assumes a (hypothetical) willingness to participate in screening that increases linearly up to age 75 and decreases linearly thereafter. The resulting triangle like age distribution of participants is similar to that found in a primary care screening initiative [59]. A complete list of the non-country-specific assumptions can be found in [36].

The effect of uncertainties in the model assumptions on the model result is tested by the following deterministic sensitivity analyses:

- Ratio of detected/not detected atrial fibrillation (base case 3:1, lower value 1.5:1, upper value 10:1).
- Epidemiology assumptions (prevalence of atrial fibrillation): lower value GBD − 20%, upper value GBD + 20% compared to the base case.
- Epidemiology assumptions (incidence of stroke): lower value GBD − 20%, upper value GBD + 20% compared to the base case.
- Cost assumptions / ratio of inpatient and outpatient treatment costs of the countries considered to the costs in Germany, lower value: index value − 20%, upper value: index value + 20% compared to the base case (see Table 2).
- Discounting: the discount factor is discounted from 1% (lower value) to 5% (upper value).
- Duration: in deviation from the remaining lifetime, the effect of shorter durations (5 /10 years) is also examined.

The other model parameters are not country specific. Uncertainties in the parameter values are therefore expected to have a similar effect as shown in the study by [36].

MS Excel 2019 (Microsoft Corp. Redmond, WA) was used for the model calculations to determine the results for the base case and deterministic sensitivity calculations.

## Results

### Base case (costs)

The cost result of the model varied strongly in the base case between the countries considered. The highest contribution of the screening from a cost perspective was observed for the model result of Switzerland (− €74.31). The lowest contributions were observed for Poland (+€19.90) and Serbia (+€33.13). Stroke costs range between €7301 (Serbia) and €45,763 (Switzerland), according to the model assumptions described above, and have the strongest influence on the respective country result. For countries whose health cost index is below the index of Germany according to the OECD analysis [35], the screening costs were adjusted accordingly. This adjustment had a beneficial effect on the cost result in the case of Greece, whose population also has a relatively high life expectancy (cost result Greece: +€5.97 per screening). Cost results for Germany (+ 5.63€ per screening) differ from the results published in [36] (− 109.96€) because of the exclusion of the risk structure compensation effect, which is specific to Germany. Base case results per country are listed in Table 5.

**Table 5:** Model base case results by country, simulated cohort

	Delta Cost [€] per participant	Delta QALY per 1000 participants	Strokes prevented per 1000 participants	ICER [€/year] (Delta cost per QALY)
Switzerland	−74.91	10.5	2.52	Scrrening dominates
UK	−6.81	12.6	3.06	
Greece	5.97	12.2	3.05	€489
Netherlands	15.44	10.1	2.48	€1529
Poland	19.90	18.7	4.43	€1064
Serbia	33.13	13.0	3.43	€2548
Germany *	5.63	14.7	3.65	€382

Simulated cohort: 65–85 years, determined by general age distribution and AF prevalence by age

*Germany: model assumptions adopted from [36] (disregarding the risk structure compensation effect)

The expected cost change correlates strongly with stroke costs (here: first year costs) (*r* = − 0.91, *p* < 0.01, *n* = 7 countries including Germany). The model results also show a significant relationship between the expected cost change and the expected remaining lifetime in the simulated cohort (*r* = − 0.77, *p* = 0.045). The influence of the other epidemiological parameters on the model result is smaller. Because of the small number of countries considered and because of the intercorrelations between possible regressors mentioned above, quantifying the influence of the model parameters by means of multiple regression analysis is not meaningful.

### Base case (effectiveness)

**Fig. 2:**
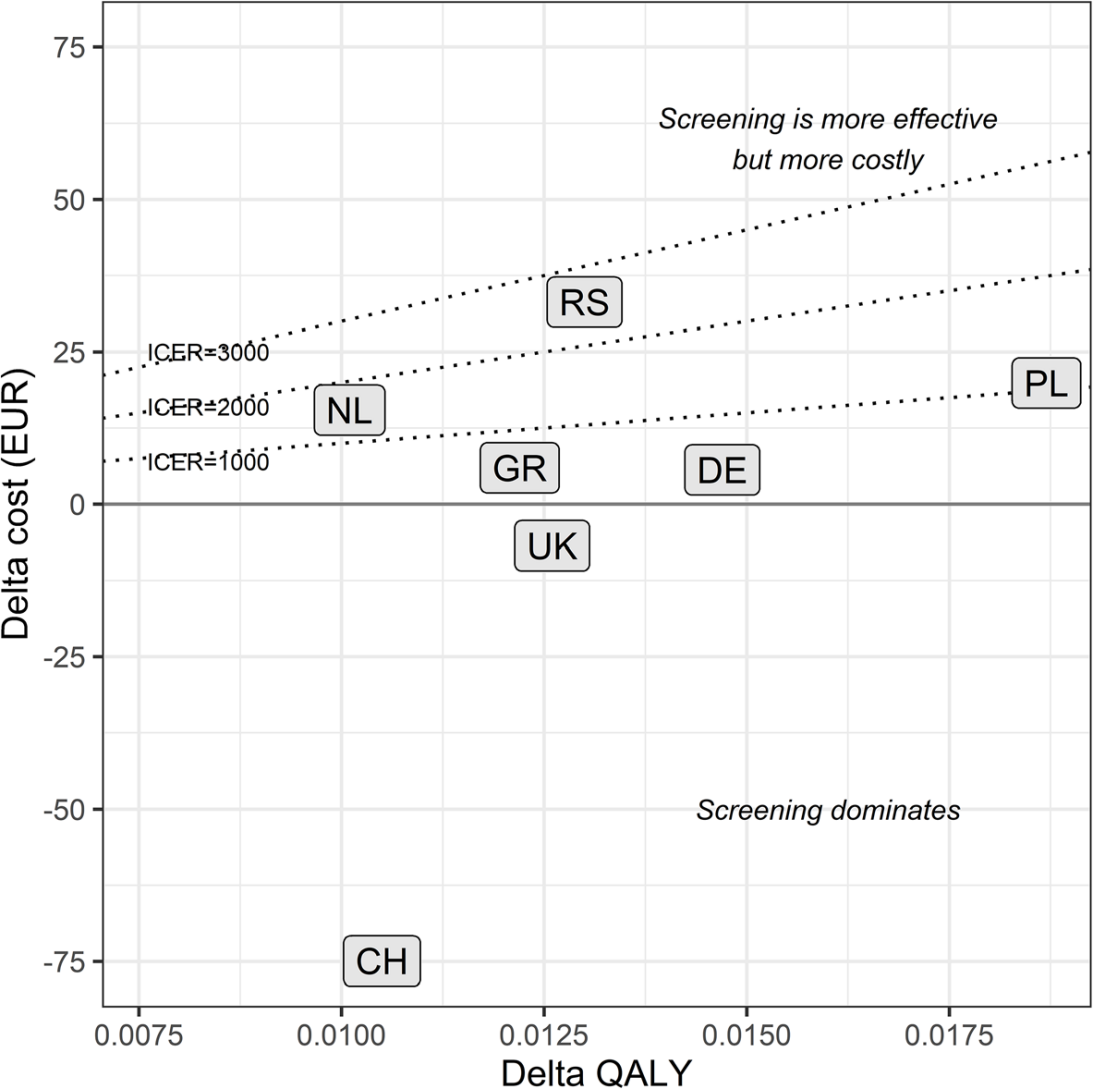
Position of base case country results in the cost-effectiveness plane. QALY: quality-adjusted life years; ICER: incremental cost-effectiveness ratio; NL: Netherlands; RS: Serbia; PL: Poland; DE: Germany; GR: Greece; UK: United Kingdom; CH: Switzerland

The effectiveness of the screening procedure (measured here as number of avoided strokes per 1000 participants) ranged from 2.5 avoided strokes (Netherlands, Switzerland) to 4.4 avoided strokes (Poland). In terms of quality-adjusted life years (QALY) gained, the results for the countries considered ranged from 0.010 (Netherlands) to 0.018 years (Poland). Figure 2 shows the average Cost/QALY position of the 6 considered countries (plus Germany) in the cost-effectiveness plane.

### Sensitivity calculations

**Fig. 3:**
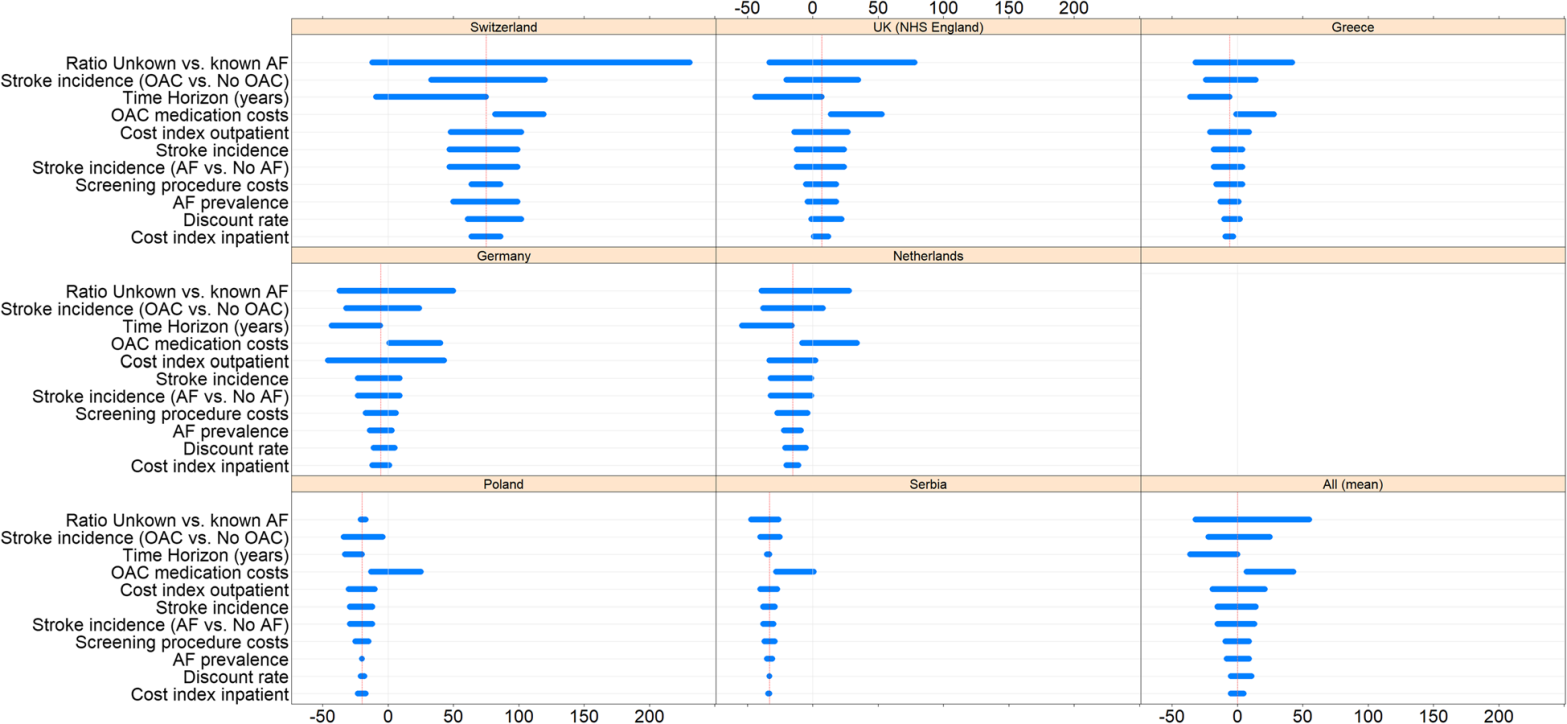
Deterministic sensitivity analysis results by country: Cost delta. Bars (horizontal): span of sensitivity analysis results; grey line (vertical): cost delta = 0 (origin); red line (vertical): base case result. Numeric results: see Table 6

Within the framework of a deterministic sensitivity analysis, the dependence of the model result on individual model parameters was examined (Fig. 3, Tables 6 and 7). As in the results of the parent model [36] the AF morbidity of the target population, in particular the proportion of patients with undiagnosed AF, has a strong influence on the model outcome. The model assumes high effectiveness of prevention caused by oral anticoagulants. The model assumption in the base case is a 70% reduction in stroke incidence in AF with OAC vs. stroke incidence without OAC. This value is based on meta-analyses by [22, 24]. A large reduction in this ratio to 56% in the sensitivity calculations leads to a significant reduction in the outcome contribution of screening.

**Table 6:** Cost Delta: Deterministic Sensitivity Analysis (by country)

Cost delta per screening	Range	Switzerland	Germany	Greece	Netherlands	Poland	Serbia	UK
Base Case		−74.91 €	5.63 €	5.97 €	15.44 €	19.90 €	33.13 €	−6.80 €
Ratio Unkown vs. known AF	[10% ... 75%]	[13 - -232 €]	[37 - -50 €]	[33 - -42 €]	[40 - -28 €]	[22–17 €]	[47–25 €]	[33 - -78 €]
Stroke incidence (OAC vs. No OAC)	[− 20%;+ 20%]	[−33 - -120 €]	[33 - -24 €]	[25 - -14 €]	[38 - -8 €]	[35–4 €]	[41–24 €]	[20 - -36 €]
Time Horizon (years)	[5 ... 20 yrs]	[10 - -75 €]	[44–5 €]	[37–6 €]	[55–15 €]	[34–19 €]	[35–32 €]	[44 - -7 €]
OAC medication costs	[− 70%;-10%]	[−81 - -119 €]	[−1 - -40 €]	[1 - -28 €]	[8 - -34 €]	[13 - -26 €]	[28 - -2 €]	[−14 - -54 €]
Cost index outpatient	[−20%;+ 20%]	[−48 - -102 €]	[47 - -44 €]	[21 - -9 €]	[33 - -2 €]	[31–9 €]	[40–26 €]	[14 - -28 €]
Stroke incidence	[− 20%;+ 20%]	[− 47 - -100 €]	[24 - -10 €]	[18 - -5 €]	[32–0 €]	[30–12 €]	[38–29 €]	[13 - -25 €]
Stroke incidence (AF vs. No AF)	[− 20%;+ 20%]	[− 47 - -100 €]	[24 - -10 €]	[18 - -5 €]	[32–0 €]	[30–12 €]	[38–29 €]	[13 - -25 €]
Screening procedure costs	[− 70%;-10%]	[−64 - -86 €]	[17 - -6 €]	[16 - -4 €]	[27–4 €]	[25–15 €]	[38–28 €]	[5 - -19 €]
AF prevalence	[−20%;+ 20%]	[− 50 - -100 €]	[15 - -3 €]	[14 - -2 €]	[22–8 €]	[20–19 €]	[35–31 €]	[5 - -18 €]
Discount rate	[0% ... 5%]	[− 60 - -102 €]	[11 - -5 €]	[11 - -3 €]	[21–4 €]	[21–17 €]	[34–33 €]	[1 - -22 €]
Cost index inpatient	[−20%;+ 20%]	[−63 - -87 €]	[13 - -1 €]	[10–2 €]	[21–10 €]	[24–16 €]	[34–32 €]	[− 1 - -13 €]

**Table 7:** Effectiveness (avoided strokes): Deterministic Sensitivity Analysis (by country)

Strokes prevented	Range	Switzerland	Germany	Greece	Netherlands	Poland	Serbia	UK
(per 1000 screening participants)								
Base Case		2.53	3.65	3.06	2.48	4.43	3.43	3.06
Ratio Unkown vs. known AF	[10% ... 75%]	[0.8–5.7]	[1.1–8.2]	[0.9–6.9]	[0.7–5.6]	[1.3–10.0]	[1.0–7.7]	[0.9–6.9]
Stroke incidence (OAC vs. No OAC)	[−20%;+ 20%]	[1.9–3.2]	[2.7–4.7]	[2.3–4.0]	[1.9–3.1]	[3.3–5.8]	[2.5–4.6]	[2.3–3.9]
AF prevalence	[−20%;+ 20%]	[2.0–3.0]	[2.9–4.4]	[2.4–3.7]	[2.0–3.0]	[3.5–5.3]	[2.7–4.1]	[2.4–3.7]
Stroke incidence	[−20%;+ 20%]	[2.2–2.8]	[3.2–4.0]	[2.7–3.3]	[2.1–2.8]	[4.0–4.7]	[3.2–3.6]	[2.7–3.4]
Stroke incidence (AF vs. No AF)	[−20%;+ 20%]	[2.2–2.8]	[3.2–4.0]	[2.7–3.3]	[2.1–2.8]	[4.0–4.7]	[3.2–3.6]	[2.7–3.4]

The change in cost indices leads to the expected results - lower unit costs increased the contribution of screening. Of note is the strong response of the model to the expected reduction in OAC costs under the Direct Oral Anticoagulants patent expiry. The mean (unweighted) contribution of screening increases from − 1 Euro to 41 Euros when the cost of OAC is reduced in the 7 countries considered by 70%. In Poland, a 30% reduction in the price of OAC results in a positive profit contribution from screening. In Serbia, a price reduction of the OAC by 70% is necessary to achieve a positive profit contribution.

The model results for Poland and Serbia show low sensitivity with respect to the model parameters under review, except for OAC costs, to changes in which the model result is most sensitive in the mentioned countries. The reason is that treatment costs in these countries are at a much lower level, while prevention costs for treatment with DOAC do not differ much from the other countries considered.

Regarding the number of avoided strokes as a measure of effectiveness, the model shows the highest sensitivity to changes in the ratio of undetected to detected AF cases. In populations with a high proportion of undetected AF cases (a ratio of 50 and 75% was simulated), the number of avoided strokes per 1000 patients screened increases from 6.2 (base case: ratio 33%) to 9.1 (ratio 50%) and 13.6 (ratio 75%) on average for all countries considered.

### Country-specific sensitivity calculations

Further sensitivity analyses for individual country-specific parameters show the high dependence of the model result on the parameter settings:

- Serbia: Based on the results of [60], the VKA share of total OAC was changed to 67%. As a result, the cost delta for Serbia decreased to €10.44.
- Netherlands: The base case the Dutch stroke incidence rate per 100.000 was 178. Vaartjes et.al [61]. identified 41,000 first-ever strokes per year in the Netherlands, resulting in an incidence rate per 100,000 of 237. The increased incidence rate reduces the cost delta for the Netherlands to €9.24.

The model reacts insensitively to changes in the discount rate. In most countries (except Switzerland), a short duration of the model after the start of prevention (5 years instead of lifetime in the sensitivity calculations) is not sufficient to compensate for the screening costs incurred at the beginning by positive cost effects from the reduced number of strokes.

### ICER results

In two of the seven countries under consideration (UK and Switzerland), the screening dominated the alternative (Fig. 3). For the remaining countries, the model base case resulted in increased effectiveness going along with additional costs for the screening alternative.

## Discussion

Our approach to simulate the success of an atrial fibrillation screening program in 6 further countries after an analysis for Germany. Applied was a common modelling framework that reflects the different epidemiologic and health economic conditions in these countries yielded several insights regarding the potential use of screening procedures from both economic and medical perspectives.

For Switzerland and the UK, screening is the dominant strategy. In the remaining 5 countries, the incremental cost-effectiveness ratio is below the relatively low value of €3000 euros per quality-adjusted life year. In Serbia, the combination of prices for drug-based stroke prophylaxis at international levels and low costs for stroke treatment in international comparison led to the low cost-effectiveness of screening compared with the seven countries. Similar results can be expected for other East or South-East European countries. The expected price reduction for direct oral anticoagulants due to the upcoming patent expiry will require a reassessment of the cost-effectiveness of screening. The sensitivity analysis showed that changes in the overall levels of stroke incidence and AF prevalence had comparably small effects on the model result. An exception is the yield ratio driven by the AF prevalence and the ratio of undetected vs. detected AF in the population. The result of the sensitivity analysis emphasizes the importance of focusing the screening on populations with high AF morbidity, for example, by preselecting participants using morbidity-based criteria such as the CHA_2_DS_2−_VASc score.

For the epidemiological parameters of interest in this study, a number of multinational epidemiological studies [32, 33, 51, 55] were identified. Their results diverge widely. With the exception of [51] these studies are limited to small prespecified groups of countries.

To the best of the authors’ knowledge, there is no comparable study comparing the cost-effectiveness of AF screening procedures in several countries within a common modelling framework. The cost-effectiveness of AF screening in European countries was evaluated in studies [62] (UK), [63] (Sweden), and [64] (Netherlands). Two HTA studies by NICE [65] and the Irish authority HIQA [66] also addressed the cost-effectiveness of screening procedures. Hobbs et.al [62]. used RCT to compare targeted screening of a target population of over 65-year-olds with usual care. Screening was performed by ECG. Screening achieved a yield rate of 1.6%, compared with 1.1% in the control arm. The associated economic model included stroke prophylaxis with VKA as DOAC were not available at the time of the study (2005). Incremental costs per additional detected AF case ranged from £ 363 to £ 4088. Aronsson et.al [63]. modelled cost-effectiveness based on the results of the STROKESTOP [57] community study, in which 75-year-olds were screened for previously undetected AF by ECG. Screening resulted in 0.0013 QALY per participant. The detection rate of previously unknown AF was 3%. The incremental cost per QALY was €4313. The Irish Health Information and Quality Authority [66] modelled the screening of the entire population aged 65 years and older in Ireland using pulse palpation and ECG with a time horizon of 25 years. Screening results in an additional cost of €73 per case, with a quality-of-life gain of 0.0034 QALY. The ICER of €20,271 per QALY is well above the result of the other studies. Jacobs et.al [64]. modeled screening of the entire Dutch population over 65 years of age using a handheld ECG device. Cost-effectiveness was reported for 25,387 patients who had previously unrecognized AF in the model outcome. For these patients, cost savings amounted to €764 and QALYs were increased by 0.27 years per case. Restricting the endpoints to new cases of screening-detected AF limits the comparability with the present study and the other cost-effectiveness studies mentioned here.

Apart from the health economic considerations presented here, the socioeconomic impact of telehealth devices needs deeper evaluation. Deprivation and social status are key prognostic factors for chronic diseases (e.g., [39, 67, 68]). The urban-rural divide influences both the level of deprivation and the outcome quality [69, 70]. Telehealth is expected to reduce inequalities and to bridge the urban-rural divide [71, 72] and can contribute to balancing regionally disparate capabilities for detecting atrial fibrillation. But, despite the widespread availability of the Internet and mobile devices, it cannot be excluded that layers of each of the targeted societies are without such access and are therefore disadvantaged. Here some further research may be necessary.

## Conclusion

This analysis confirms overall the positive effects of a systematic screening for atrial fibrillation with a photopethysmographic procedure. Quality adjusted life-years can be gained. Costs may be saved in some countries. The model results showed a strong dependence on the country-specific costs for stroke treatment. The use of the investigated screening method is close to cost-neutral or cost-reducing in the Western European countries and Greece. In countries with lower price levels, cost increases due to AF screening are to be expected. Lower costs of anticoagulation, which are expected due to the upcoming patent expiry of direct anticoagulants, will have a positive effect on the cost result. The outcomes should be viewed individually for each investigated country.

## Limitations

Sensitivity analyses show a high dependence of the result on the assumptions regarding the effectiveness of stroke prevention by OAC. Effectiveness parameters need to be supported by further literature searches, ideally by a meta-analysis.

The results of the study reflect a payor perspective in the countries of consideration. Only direct costs were included, disregarding informal and other indirect costs related to stroke events. The assumptions to determine country-specific costs made in this study appear conclusive but need to be verified by further empirical studies.

The stroke treatment costs were shown to have a large influence on the model result. The estimates employed here are broad approximations based on a German source [40] and extrapolated to other countries by health price indexes. A thorough cost analysis would require a detailed analysis of the country-specific stroke costs within a common analysis framework, as laid out by [1] or [73].

In concordance with the ESC guidelines [23] for AF screening, the model was limited to populations aged 65 years or older. The Eastern European countries considered here show excessive stroke incidence rates in subpopulations aged between 55 and 65 years. An extension of the modelling to these age groups is likely to reflect the epidemiological reality of these countries in a more realistic way.

## Supplementary Information

**Additional file 1.** Supplement: Derivation of pricing assumptions (VKA, DOAC, hospital costs).

## Data Availability

All data generated or analyzed for the economic model are included in this published article.

## References

[1] Luengo-Fernandez R, Violato M, Candio P, Leal J (2020). Economic burden of stroke across Europe: A population-based cost analysis. Eur Stroke J.

[2] Stevens E, Emmett E, Wang Y, McKevitt C, Wolfe C (2017). The Burden of Stroke in Europe: The challenge for policy makers. Stroke Alliance for Europe.

[3] Truelsen T, Piechowski-Jóźwiak B, Bonita R, Mathers C, Bogousslavsky J, Boysen G (2006). Stroke incidence and prevalence in Europe: a review of available data. Eur J Neurol.

[4] Wafa HA, Wolfe CDA, Emmett E, Roth GA, Johnson CO, Wang Y (2020). Burden of stroke in Europe: thirty-year projections of incidence, prevalence, deaths, and disability-adjusted life years. Stroke.

[5] Krijthe BP, Kunst A, Benjamin EJ, Lip GYH, Franco OH, Hofman A, Witteman JCM, Stricker BH, Heeringa J (2013). Projections on the number of individuals with atrial fibrillation in the European Union, from 2000 to 2060. Eur Heart J.

[6] Fuster V, Rydén LE, Cannom DS, Crijns HJ, Curtis AB, Ellenbogen KA, Halperin JL, Le Heuzey J-Y, Kay GN, Lowe JE, Olsson SB, Prystowsky EN, Tamargo JL, Wann S, Smith SC, Jacobs AK, Adams CD, Anderson JL, Antman EM, Hunt SA, Nishimura R, Ornato JP, Page RL, Riegel B, Priori SG, Blanc J-J, Budaj A, Camm AJ, Dean V, Deckers JW (2006). ACC/AHA/ESC 2006 guidelines for the management of patients with atrial fibrillation—executive summary: a report of the American College of Cardiology/American Heart Association Task Force on Practice Guidelines and the European Society of Cardiology Committee for Practice Guidelines (Writing Committee to Revise the 2001 Guidelines for the Management of Patients With Atrial Fibrillation). J Am Coll Cardiol.

[7] Perings C, Hennersdorf M, Vester EG, Weirich J, Strauer BE (1998). Pathophysiologie, Epidemiologie und Komplikationen des Vorhofflimmerns. Internist.

[8] Benjamin EJ, Wolf PA, D’Agostino RB, Silbershatz H, Kannel WB, Levy D (1998). Impact of atrial fibrillation on the risk of death: the Framingham heart study. Circulation.

[9] Kannel W, Wolf P, Benjamin E, Levy D (1998). Prevalence, incidence, prognosis, and predisposing conditions for atrial fibrillation: population-based estimates 11Reprints are not available. Am J Cardiol.

[10] Thrall G, Lane D, Carroll D, Lip GYH (2006). Quality of life in patients with atrial fibrillation: a systematic review. Am J Med.

[11] Wang TJ, Larson MG, Levy D, Vasan RS, Leip EP, Wolf PA, D’Agostino RB, Murabito JM, Kannel WB, Benjamin EJ (2003). Temporal relations of atrial fibrillation and congestive heart failure and their joint influence on mortality: the Framingham heart study. Circulation.

[12] Bramlage K, Thoenes M, Tebbe U, Bramlage P, Wasem J (2013). Vorhofflimmern in Deutschland-- Epidemiologie, Diagnostik, Therapie und Kosten [Atrial fibrillation in Germany--epidemiology, diagnosis, treatment and costs]. Med Monatsschr Pharm.

[13] Benkert D, Theres H, Wasem J, Aidelsburger P (2009). Direkte Kosten in der Diagnostik und Behandlung von Patienten mit symptomatischem Vorhofflimmern in Deutschland. Pharmacoeconomics-Ger-Res-Articles.

[14] Reinhold T, Rosenfeld S, Müller-Riemenschneider F, Willich SN, Meinertz T, Kirchhof P, Brüggenjürgen B (2012). Patienten mit Vorhofflimmern in Deutschland. Charakteristika, Ressourcenverbrauch und Kosten. Herz.

[15] Go AS, Hylek EM, Phillips KA, Chang Y, Henault LE, Selby JV, Singer DE (2001). Prevalence of diagnosed atrial fibrillation in adults: national implications for rhythm management and stroke prevention: the AnTicoagulation and risk factors in atrial fibrillation (ATRIA) study. JAMA.

[16] Andersson T, Magnuson A, Bryngelsson I-L, Frøbert O, Henriksson KM, Edvardsson N, Poçi D (2013). All-cause mortality in 272,186 patients hospitalized with incident atrial fibrillation 1995-2008: a Swedish nationwide long-term case-control study. Eur Heart J.

[17] Nieuwlaat R, Capucci A, Camm AJ, Olsson SB, Andresen D, Davies DW, Cobbe S, Breithardt G, Le Heuzey J-Y, Prins MH, Lévy S, Crijns HJGM (2005). Atrial fibrillation management: a prospective survey in ESC member countries: the euro heart survey on atrial fibrillation. Eur Heart J.

[18] Ruigómez A, Johansson S, Wallander MA, Rodríguez LAG (2002). Incidence of chronic atrial fibrillation in general practice and its treatment pattern. J Clin Epidemiol.

[19] Savelieva I, Bajpai A, Camm AJ (2007). Stroke in atrial fibrillation: update on pathophysiology, new antithrombotic therapies, and evolution of procedures and devices. Ann Med.

[20] Spratt N, Wang Y, Levi C, Ng K, Evans M, Fisher J (2003). A prospective study of predictors of prolonged hospital stay and disability after stroke. J Clin Neurosci.

[21] van Walraven C, Hart RG, Singer DE, Laupacis A, Connolly S, Petersen P, Koudstaal PJ, Chang Y, Hellemons B (2002). Oral anticoagulants vs aspirin in nonvalvular atrial fibrillation: an individual patient meta-analysis. JAMA.

[22] Hart RG, Benavente O, McBride R, Pearce LA (1999). Antithrombotic therapy to prevent stroke in patients with atrial fibrillation: a meta-analysis. Ann Intern Med.

[23] Kirchhof P, Benussi S, Kotecha D, Ahlsson A, Atar D, Casadei B, Castella M, Diener H-C, Heidbuchel H, Hendriks J, Hindricks G, Manolis AS, Oldgren J, Popescu BA, Schotten U, van Putte B, Vardas P (2016). 2016 ESC guidelines for the management of atrial fibrillation developed in collaboration with EACTS. Eur Heart J.

[24] López-López JA, Sterne JAC, Thom HHZ, Higgins JPT, Hingorani AD, Okoli GN, Davies PA, Bodalia PN, Bryden PA, Welton NJ, Hollingworth W, Caldwell DM, Savović J, Dias S, Salisbury C, Eaton D, Stephens-Boal A, Sofat R (2017). Oral anticoagulants for prevention of stroke in atrial fibrillation: systematic review, network meta-analysis, and cost effectiveness analysis. BMJ (Clinical research ed.).

[25] Camm AJ, Kirchhof P, Lip GYH, Schotten U, Savelieva I, Ernst S, van Gelder IC, Al-Attar N, Hindricks G, Prendergast B, Heidbuchel H, Alfieri O, Angelini A, Atar D, Colonna P, de Caterina R, de Sutter J, Goette A, Gorenek B, Heldal M, Hohloser SH, Kolh P, Le Heuzey J-Y, Ponikowski P, Rutten FH (2010). Guidelines for the management of atrial fibrillation: the task force for the management of atrial fibrillation of the European Society of Cardiology (ESC). Eur Heart J.

[26] Connolly SJ, Ezekowitz MD, Yusuf S, Eikelboom J, Oldgren J, Parekh A, Pogue J, Reilly PA, Themeles E, Varrone J, Wang S, Alings M, Xavier D, Zhu J, Diaz R, Lewis BS, Darius H, Diener H-C, Joyner CD, Wallentin L (2009). Dabigatran versus warfarin in patients with atrial fibrillation. N Engl J Med.

[27] Granger CB, Alexander JH, McMurray JJV, Lopes RD, Hylek EM, Hanna M, Al-Khalidi HR, Ansell J, Atar D, Avezum A, Bahit MC, Diaz R, Easton JD, Ezekowitz JA, Flaker G, Garcia D, Geraldes M, Gersh BJ, Golitsyn S, Goto S, Hermosillo AG, Hohnloser SH, Horowitz J, Mohan P, Jansky P, Lewis BS, Lopez-Sendon JL, Pais P, Parkhomenko A, Verheugt FWA (2011). Apixaban versus warfarin in patients with atrial fibrillation. N Engl J Med.

[28] Rocket AF, Study Investigators (2010). Rivaroxaban-once daily, oral, direct factor Xa inhibition compared with vitamin K antagonism for prevention of stroke and Embolism Trial in Atrial Fibrillation: rationale and design of the ROCKET AF study. Am Heart J.

[29] Brasier N, Raichle CJ, Dörr M, Becke A, Nohturfft V, Weber S, Bulacher F, Salomon L, Noah T, Birkemeyer R, Eckstein J (2019). Detection of atrial fibrillation with a smartphone camera: first prospective, international, two-centre, clinical validation study (DETECT AF PRO). Europace.

[30] Koenig N, Seeck A, Eckstein J, Mainka A, Huebner T, Voss A, Weber S (2016). Validation of a new heart rate measurement algorithm for fingertip recording of video signals with smartphones. Telemed J E Health.

[31] Institute for Health Metrics and Evaluation (IHME) (2018). Findings from the global burden of disease study 2017.

[32] Ceornodolea AD, Bal R, Severens JL (2017). Epidemiology and management of atrial fibrillation and stroke: review of data from four European countries. Stroke Research and Treatment.

[33] Zhang Y, Chapman A-M, Plested M, Jackson D, Purroy F (2012). The incidence, prevalence, and mortality of stroke in France, Germany, Italy, Spain, the UK, and the US: a literature review. Stroke Res Treat.

[34] Huber M (2016). International comparisons of prices and volumes in health care among OECD countries.

[35] Lorenzoni L, Koechlin F (2017). International Comparisons of Health Prices and Volumes: New Findings; Paris, France: Organisation for Economic Co-operation and Development (OECD).

[36] Birkemeyer R, Müller A, Wahler S, von der Schulenburg J-M (2020). A cost-effectiveness analysis model of Preventicus atrial fibrillation screening from the point of view of statutory health insurance in Germany. Health Econ Rev.

[37] Hindricks G, Potpara T, Dagres N, Arbelo E, Bax JJ, Blomström-Lundqvist C, Boriani G, Castella M, Dan G-A, Dilaveris PE, Fauchier L, Filippatos G, Kalman JM, La Meir M, Lane DA, Lebeau J-P, Lettino M, Lip GYH, Pinto FJ, Thomas GN, Valgimigli M, van Gelder IC, van Putte BP, Watkins CL (2021). 2020 ESC guidelines for the diagnosis and management of atrial fibrillation developed in collaboration with the European Association for Cardio-Thoracic Surgery (EACTS): the task force for the diagnosis and management of atrial fibrillation of the European Society of Cardiology (ESC) developed with the special contribution of the European heart rhythm association (EHRA) of the ESC. Eur Heart J.

[38] Wachter R, Gröschel K, Gelbrich G, Hamann GF, Kermer P, Liman J, Seegers J, Wasser K, Schulte A, Jürries F, Messerschmid A, Behnke N, Gröschel S, Uphaus T, Grings A, Ibis T, Klimpe S, Wagner-Heck M, Arnold M, Protsenko E, Heuschmann PU, Conen D, Weber-Krüger M (2017). Holter-electrocardiogram-monitoring in patients with acute ischaemic stroke (find-AF RANDOMISED ): an open-label randomised controlled trial. Lancet Neurol.

[39] Wade V, Stocks N (2017). The use of telehealth to reduce inequalities in cardiovascular outcomes in Australia and New Zealand: a critical review. Heart Lung Circ.

[40] Kolominsky-Rabas PL, Heuschmann PU, Marschall D, Emmert M, Baltzer N, Neundörfer B, Schöffski O, Krobot KJ (2006). Lifetime cost of ischemic stroke in Germany: results and national projections from a population-based stroke registry: the Erlangen stroke project. Stroke.

[41] Mozaffarian D, Benjamin EJ, Go AS, Arnett DK, Blaha MJ, Cushman M, de Ferranti S, Després J-P, Fullerton HJ, Howard VJ, Huffman MD, Judd SE, Kissela BM, Lackland DT, Lichtman JH, Lisabeth LD, Liu S, Mackey RH, Matchar DB, McGuire DK, Mohler ER, Moy CS, Muntner P, Mussolino ME, Nasir K, Neumar RW, Nichol G, Palaniappan L, Pandey DK, Reeves MJ (2015). Heart disease and stroke statistics--2015 update: a report from the American Heart Association. Circulation.

[42] Boriani G, Proietti M, Laroche C, Fauchier L, Marin F, Nabauer M, Potpara T, Dan G-A, Kalarus Z, Diemberger I, Tavazzi L, Maggioni AP, Lip GYH (2018). Contemporary stroke prevention strategies in 11 096 European patients with atrial fibrillation: a report from the EURObservational research Programme on atrial fibrillation (EORP-AF) long-term general registry. Europace.

[43] Centrala Narodowego Funduszu Zdrowia (2019). Departament Analiz I Strategii: Ischemic Stroke (Udar niedokrwienny mózgu).

[44] Buisman LR, Tan SS, Nederkoorn PJ, Koudstaal PJ, Redekop WK (2015). Hospital costs of ischemic stroke and TIA in the Netherlands. Neurology.

[45] Kritikou P, Spengos K, Zakopoulos N, Tountas Y, Yfantopoulos J, Vemmos K (2016). Resource utilization and costs for treatment of stroke patients in an acute stroke unit in Greece. Clin Neurol Neurosurg.

[46] Krejczy M (2013). Kosten-Effektivitätsanalyse direkter oraler Antikoagulantien im Vergleich zu Warfarin bei Patienten mit Vorhofflimmern. Übertragung der Studienergebnisse auf das deutsche Gesundheitssystem.

[47] Friberg L, Rosenqvist M, Lip GYH (2012). Evaluation of risk stratification schemes for ischaemic stroke and bleeding in 182 678 patients with atrial fibrillation: the Swedish atrial fibrillation cohort study. Eur Heart J.

[48] Chatterjee S, Sardar P, Biondi-Zoccai G, Kumbhani DJ (2013). New oral anticoagulants and the risk of intracranial hemorrhage: traditional and Bayesian meta-analysis and mixed treatment comparison of randomized trials of new oral anticoagulants in atrial fibrillation. JAMA Neurol.

[49] INEK. Institut für Entgeltwesen im Krankenhaus: Fallpauschalen-Katalog 2020. Siegburg: InEK; 2020. [[https://www.g-drg.de/aG-DRG-System_2020/Fallpauschalen-Katalog/Fallpauschalen-Katalog_2020]. [Last accessed 01 Sep 2021]

[50] Bronnum-Hansen H, Davidsen M, Thorvaldsen P (2001). Long-term survival and causes of death after stroke. Stroke.

[51] Johnson CO, Nguyen M, Roth GA, Nichols E, Alam T, Abate D, Abd-Allah F, Abdelalim A, Abraha HN, NME A-R, Adebayo OM, Adeoye AM, Agarwal G, Agrawal S, Aichour AN, Aichour I, MTE A, Alahdab F, Ali R, Alvis-Guzman N, Anber NH, Anjomshoa M, Arabloo J, Arauz A, Ärnlöv J, Arora A, Awasthi A, Banach M, Barboza MA, Barker-Collo SL (2019). Global, regional, and national burden of stroke, 1990–2016: a systematic analysis for the Global Burden of Disease Study 2016. Lancet Neurol.

[52] Heuschmann P, Busse O, Wagner M, Endres M, Villringer A, Röther J, Kolominsky-Rabas P, Berger K (2010). Schlaganfallhäufigkeit und Versorgung von Schlaganfallpatienten in Deutschland. Akt Neurol.

[53] King D, Wittenberg R, Patel A, Quayyum Z, Berdunov V, Knapp M (2020). The future incidence, prevalence and costs of stroke in the UK. Age Ageing.

[54] Wilke T, Groth A, Mueller S, Pfannkuche M, Verheyen F, Linder R, Maywald U, Bauersachs R, Breithardt G (2013). Incidence and prevalence of atrial fibrillation: an analysis based on 8.3 million patients. Europace..

[55] Zoni-Berisso M, Lercari F, Carazza T, Domenicucci S (2014). Epidemiology of atrial fibrillation: European perspective. Clin Epidemiol.

[56] National Cardiovascular Intelligence Network: Atrial Fibrillation prevalence estimates. London: Public Health England; 2020. [https://www.gov.uk/government/publications/atrial-fibrillation-prevalence-estimates-for-local-populations]. [Last accessed Sep 1st, 2021].

[57] Svennberg E, Engdahl J, Al-Khalili F, Friberg L, Frykman V, Rosenqvist M (2015). Mass screening for untreated atrial fibrillation: the STROKESTOP study. Circulation.

[58] Lowres N, Neubeck L, Redfern J, Freedman SB (2013). Screening to identify unknown atrial fibrillation. A systematic review. Thromb Haemost.

[59] Kaasenbrood F, Hollander M, Rutten FH, Gerhards LJ, Hoes AW, Tieleman RG (2016). Yield of screening for atrial fibrillation in primary care with a hand-held, single-lead electrocardiogram device during influenza vaccination. Europace.

[60] Potpara TS, Dan G-A, Trendafilova E, Goda A, Kusljugic Z, Manola S, Music L, Musetescu R, Badila E, Mitic G, Paparisto V, Dimitrova ES, Polovina MM, Petranov SL, Djergo H, Loncar D, Bijedic A, Brusich S, Lip GYH (2016). Stroke prevention in atrial fibrillation and ‘real world’ adherence to guidelines in the Balkan region: the BALKAN-AF survey. Sci Rep.

[61] Vaartjes I, Reitsma JB, de Bruin A, Berger-van Sijl M, Bos MJ, Breteler MMB, Grobbee DE, Bots ML (2008). Nationwide incidence of first stroke and TIA in the Netherlands. Eur J Neurol.

[62] Hobbs FD, Fitzmaurice DA, Mant J, Murray E, Jowett S, Bryan S, Raftery J, Davies M, Lip G (2005). A randomised controlled trial and cost-effectiveness study of systematic screening (targeted and total population screening) versus routine practice for the detection of atrial fibrillation in people aged 65 and over. The SAFE study. Health Technol Assess.

[63] Aronsson M, Svennberg E, Rosenqvist M, Engdahl J, Al-Khalili F, Friberg L, Frykman-Kull V, Levin LÅ (2015). Cost-effectiveness of mass screening for untreated atrial fibrillation using intermittent ECG recording. Europace..

[64] Jacobs MS, Kaasenbrood F, Postma MJ, van Hulst M, Tieleman RG (2018). Cost-effectiveness of screening for atrial fibrillation in primary care with a handheld, single-lead electrocardiogram device in the Netherlands. Europace.

[65] Welton NJ, McAleenan A, Thom HH, Davies P, Hollingworth W, Higgins JP, Okoli G, Sterne JA, Feder G, Eaton D, Hingorani A, Fawsitt C, Lobban T, Bryden P, Richards A, Sofat R (2017). Screening strategies for atrial fibrillation: a systematic review and cost-effectiveness analysis. Health Technol Assess (Winchester, England).

[66] Moran P, Teljeur C, Harrington P, Ryan M (2015). Health technology assessment (HTA) of a national screening programme for atrial fibrillation in primary care.

[67] Weir NU, Gunkel A, McDowall M, Dennis MS (2005). Study of the relationship between social deprivation and outcome after stroke. Stroke.

[68] Wahler S, Koll C, Wahler E, Müller A (2019). PDB72 deprivation - Main cause of diabetic foot ulcer in Germany. Value Health.

[69] Loccoh EC, Joynt Maddox KE, Wang Y, Kazi DS, Yeh RW, Wadhera RK (2022). Rural-urban disparities in outcomes of myocardial infarction, heart failure, and stroke in the United States. J Am Coll Cardiol.

[70] O’Neal WT, Sandesara PB, Kelli HM, Venkatesh S, Soliman EZ (2018). Urban-rural differences in mortality for atrial fibrillation hospitalizations in the United States. Heart Rhythm.

[71] Bagchi AD (2019). Expansion of telehealth across the rural–urban continuum. State Local Govern Rev.

[72] Dvoryashina M, Tarasenko E (2021). Inclusion, diversity or disparity in telehealth during the Covid-19 pandemic. IFAC-PapersOnLine.

[73] Epstein D, Mason A, Manca A (2008). The hospital costs of care for stroke in nine European countries. Health Econ.

