# Supplementary material for: Cost-effectiveness of a photopethysmographic procedure for screening for atrial fibrillation in 6 European countries
Source: Health Econ Rev. 2022 Feb 26;12:17. doi: 10.1186/s13561-022-00362-2 (PMC8882287; doi:10.1186/s13561-022-00362-2)
Supplement: Supplementary file 1 — Additional file 1. Supplement: Derivation of pricing assumptions (VKA, DOAC, hospital costs). [file 13561_2022_362_MOESM1_ESM.docx]

**Supplement: Derivation of pricing assumptions (VKA, DOAC, hospital costs)**

**VKA, DOAC.** Pharmacy retail prices for VKA and DOAC per daily dose were obtained from national health system catalogs for Greece, Serbia, the Netherlands, and England/Wales (NHS). For Poland and Switzerland, online sources were accessed. For Germany, a publication from the National Association of Statutory Health Insurance Physicians is available (Table 1). The package prices were divided by the daily doses (DDD) of the oral anticoagulants [1] and converted into daily costs.

The shares of the alternative OAC drugs within the indication of stroke prevention is not known. To support the assumptions of the model, figures for the total consumption of OAC in DDD were used, which are regularly published for Germany [2–4]. Data for Germany show an increase in the DDD share of DOAC between 2015 and 2019 from 37% to 63%. Similar results are available for the Netherlands [5]. The Balkan AF survey [6] showed in a much higher share of VKA in Serbia (67%). A European-wide registry (EORP, [7]) with patients enrolled between 2013 and 2016 reported a VKA proportion of 35% for new AF patients. The assumption of a VKA share of 29% corresponds to the model parameters in [8] and extends the trend of decreasing VKA shares. The share of individual DOAC drugs has been adopted from [2]. Multinational research [9] suggests an increasing proportion of Apixaban and a decreasing proportion of Dabigatran. The synthesis of the daily costs in model parameters can be found in Table 2.

**Stroke treatment:** Codes and costs for acute ischemic stroke treatments were extracted from the national DRG catalogs [10–16] (Table 3). DRGs were categorized as low, moderate, and high complexity cases. According to the German DRG statistics [17], 22% of cases belong to the DRGs categorized as mild complexity, 72% of cases to the DRGs categorized as moderate complexity, and 6% of cases to the DRG group with high complexity. This weighting was adopted for the other countries considered (Table 4), unless information from alternative sources was available:

- Poland [14]: Total national spending 2018 for the four stroke DRGs was 630.4 mln zloty. 73.121 patients were hospitalized (JGP A48 – 60.8%, A49 – 16.8%; A50 – 8.9%; A51 – 13.8%) . Average spending per case was 8.621 zl (1.983 €)
- Greece [18] : 2.864 €. Data collected from 764 patients in an academic hospital in Athens.
- Netherlands [19]: 5.328 €. Average inpatient costs of 35,903 ischemic stroke patients in the Netherlands 2012.

The weighted mean case costs enter the model as the "Inpatient Cost Index".

**Table 1: Sources for VKA / NOAC Prices**

| **DDD amount [1]** | Marcumar / Warfarin | Apixaban (Eliquis®) | Dabigatran (Pradaxa®) | Rivaroxaban (Xarelto®) |
| --- | --- | --- | --- | --- |
| Starting dose | MARCUMAR [20]: 3mg Phenprocoumon 1-2 tablets (DDD: 4,5 mg)  COUMADIN: 5 mg Warfarin | 5 mg twice daily | 150 mg/110 mg twice daily | 20 mg [21] |

| **Germany** | Sources: [22] (lowest import price); online pharmacy prices: DocMorris Apotheke [23] | | | |
| --- | --- | --- | --- | --- |
| Online pharmacy, retail price | AVP Coumadin: PZN **00245546** 100 tablets, 5 mg each: 22.96 € Price/DDD: 0,23 €/DDD Assumption: 5 mg/DDD AVP Marcumar MEDA:  PZN **05541338** 98 Tablets, 3 mg each: 25.45 € Price/DDD: 0.39 €/DDD Assumption: 4.5 mg / DDD | AVP Eliquis: **PZN 0647809** 200 tablets, 5 mg each: 260.74 € Price/DDD: 2.61 € Assumption DDD: 10mg | AVP Pradaxa: PZN **10218355** 100 tablets, 110 mg each: 168.33 Price/DDD: 3.37 € Assumption DDD: 220 mg AVP Pradaxa: PZN 10249781 60 Tablets zu 150 mg: 104.59 € Price/DDD: 3.49 € Assumption DDD: 300 mg | AVP Xarelto: PZN **08461433**  98 tablets, 20 mg each: 320.80 Price/DDD: 3.27 €/DDD Assumption DDD: 20 mg |
| Cost per DDD [22] |  | 2.65 €/DDD | 3.07 €/DDD | 3.01 €/DDD |

| **Switzerland** | Online pharmacy prices [24]; Exchange rate: 1 CHF = 0.94 € (June 2020) | | | | |
| --- | --- | --- | --- | --- | --- |
| Online pharmacy, retail price | Marcoumar tablets 3mg 100 Tablets: 20.80 CHF  Price/DDD: 0.31 CHF 0.33 €/DDD,  Assumption DDD: 4.5 mg  Warfarin not available | Eliquis film tablets 5mg 168 Tablets, 260.20 CHF Price pro DDD: 3.10 CHF 3.30 €/DDD; DDD: 10 mg | Pradaxa capsules 110mg  60 Tablets 105.00 CHF Price/DDD: 3.50 CHF 3.72 €/DDD; DDD: 220 mg  Pradaxa capsules 150mg  60 Tablets 105.00 CHF Price/DDD: 3.50 CHF 3.72 €/DDD; DDD: 300 mg | Xarelto film tablets 20mg 98 Tablets, 313.50 CHF Price/DDD: 3.20 CHF 3.40 €/DDD; DDD: 20 mg |  |

| **Greece** | Online pharmacy prices: [25]  Ministry of Health: Bulletin D3 (a) 87611 / 13-12-2019 [26] | | | |
| --- | --- | --- | --- | --- |
|  | Marcumar / Warfarin | Apixaban (Eliquis®) | Dabigatran (Pradaxa®) | Rivaroxaban (Xarelto®) |
| Online pharmacy,  retail price | Code 07524.01.01  20x5mg PANWARFIN 2.33 € 0.12 €/tablet 0.12 €/DDD  (Assumption DDD=5 mg) | Code 29892.02.08  28x5mg Eliquis 39.03 € 2.78 €/DDD | Code 28235.02.04  60x110 mg Pradaxa 68.83 €  2.28 €/DDD  Code 28235.03.05  60x150 mg Pradaxa 69.35 €  2.32 €/DDD | Code 28569.03.05  100x20mg Xarelto 335.91 € 3.35 €/tablet 3.35 €/DDD (assumption:  20 mg/DDD) |
| Government source,  retail price |  | 28x5mg Eliquis 39.03 € | 60x110 mg Pradaxa 68.83 €  60x150 mg Pradaxa 69.35 € | 28x20mg Blister Xarelto 64.69 € 2.31 €/DDD |

| **Serbia** | RFZO: National Health Insurance Fund, Catalogue B, 28.3.2020 [27] Exchange rate 0.0085 DIN/€ (June 2020) | | | |
| --- | --- | --- | --- | --- |
|  | Marcumar / Warfarin | Apixaban (Eliquis®) | Dabigatran (Pradaxa®) | Rivaroxaban (Xarelto®) |
| RFZO, wholesale price | RFZO 1063115 (Lista A) FARIN blister, 30 tablets (5 mg)  Price per package 115 DIN  Price per DDD (7.5 mg) 5.75 DIN  0.033 €/DDD; DDD: 5 mg | RFZO Code 1068026  blister, 60 tablets (2.5 mg)  Price per package 6388,20 DIN  Price per DDD (10 mg) 425.88 DIN; 3.62 €/DDD; DDD: 10 mg | RFZO Code 1069614  blister, 30 tablets (110 mg)  Price per package 3148.8 DIN  Price per DDD (10 mg) 286.25 DIN; 2.43 €/DDD; DDD: 300 mg | RFZO Code 1069600  blister, 10 tablets (10 mg)  Price per package 2138.6 DIN  Price per DDD (10 mg) 213.86 DIN; 3.64 €/DDD; DDD: 20 mg |

| **Poland** | Patient information “Medycyna praktyczna, baza leków” [28]  Exchange rate 1zl = 0,23 € (June 2020) | | | |
| --- | --- | --- | --- | --- |
|  | Marcumar / Warfarin | Apixaban (Eliquis®) | Dabigatran (Pradaxa®) | Rivaroxaban (Xarelto®) |
| Medycyna praktyczna, Baza leków (retail price) | Warfin 5 mg, 100 tablets 19,08 - 28,44 zl (highest price mentioned) Price per DDD: 0.29 – 0.43 zl 0,044 - 0,065 €/DDD | Eliquis 5 mg, 60 film tablets 358.49 zł  Price per DDD: 11.95 zł 2.75 €/DDD; DDD: 10 mg | Pradaxa 110 mg, 60 capsules, 336.49 zł  Price per DDD: 11.22 zł 2.58 €/DDD; DDD: 220 mg | Xarelto 20 mg, 28 film tables, 359.99 zł Price per DDD: 12.86 zł 2.96 €/DDD; DDD: 20 mg |

| **England / Wales  (NHS – UK)** | NHS Drug Tariff, June 2020, part VIIIA [28]  Exchange rate: 1 UKP = 1.11 € (June 2020) | | | |
| --- | --- | --- | --- | --- |
|  | Marcumar / Warfarin | Apixaban (Eliquis®) | Dabigatran (Pradaxa®) | Rivaroxaban (Xarelto®) |
| NHS: Basic price of drug | Warfarin 5mg tablets  28 tablets: 1.08 UKP  Price/DDD: 0.039 UKP 0.043 €/DDD, Assumption DDD: 5 mg  Marcumar not available | Apixaban 5mg tablets  56 tablets, 53.20 UKP Price per DDD: 1.90 UKP 2.11 €/DDD; DDD: 10 mg | Dabigatran etexilate 110mg capsules 60 capsules, 51.00 UKP Price/DDD: 1.70 UKP ~ 1.89 €/DDD; DDD: 220 mg  Dabigatran etexilate 150mg capsules 60 capsules, 51.00 UKP Price/DDD: 1.70 UKP ~ 1.89 €/DDD; DDD: 300 mg | Xarelto 10mg tablets  30 tablets, 54.00 UKP Price per DDD: 3.60 UKP 3.24 €/DDD; DDD: 20 mg |

| **The Netherlands** | Farmacotherapeutisch Kompas: Kostenoverzicht direct werkende orale anticoagulantia, Zorginstituut Nederland [29] | | | |
| --- | --- | --- | --- | --- |
|  | Marcumar / Warfarin | Apixaban (Eliquis®) | Dabigatran (Pradaxa®) | Rivaroxaban (Xarelto®) |
| Zoorginstitut, kostenoverzicht | Warfarin not mentioned;  Marcoumar tablet (3mg) Per day: 0.08 € (3 mg/DDD) 0.12 €/DDD; DDD: 4.5 mg | Xarelto, tablet (10 mg)  Per day: 4.48 € (DDD: 20 mg)  2.24 €/DDD; DDD: 10 mg | Pradaxa, capsule, 110 mg Per day: 3.32 € (DDD: 300 mg)  2.43 €/DDD; DDD: 220 mg  Pradaxa, capsule, 110 mg Per day: 2.44 € (DDD: 300 mg)  2.44 €/DDD; DDD: 220 mg | ELIQUIS, TABLET 5MG  Per day: 2.25 € (DDD: 10 mg)  4.50 €/DDD; DDD: 20 mg |

**Table 2: VKA/NOAC prices: synthesis**

| **€ / daily dosage** | **Germany** | **Switzerland** | **Greece** | **Netherlands** | **Poland** | **Serbia** | **UK (NHS England)** |
| --- | --- | --- | --- | --- | --- | --- | --- |
| Warfarin | 0.23 € |  | 0.12 € | 0.12 € | 0.05 € | 0.03 € | 0.04 € |
| Marcumar | 0.39 € | 0.33 € |  |  |  |  |  |
|  |  |  |  |  |  |  |  |
| Apixaban | 2.65 € | 3.30 € | 2.78 € | 2.24 € | 2.75 € | 3.62 € | 2.11 € |
| Dabigatran 110 | 3.07 € | 3.72 € | 2.28 € | 2.43 € | 2.58 € | 2.43 € | 1.89 € |
| Dabigatran 150 |  | 3.50 € | 2.32 € | 2.44 € |  |  | 1.89 € |
| Rivaroxaban | 3.01 € | 3.40 € | 2.31 € | 4.50 € | 2.96 € | 3.64 € | 3.24 € |
| **Weights (assumption)** |  |  |  |  |  |  |  |
| **VKA** | **29%** | **29%** | **29%** | **29%** | **33%** | **29%** | **29%** |
| **DOAC** | **71%** | **71%** | **71%** | **71%** | **67%** | **71%** | **71%** |
| Apixaban | 27.0% | 27.0% | 27.0% | 27.0% | 14.0% | 27.0% | 27.0% |
| Dabiagtran | 7.1% | 7.1% | 7.1% | 7.1% | 27.0% | 7.1% | 7.1% |
| Rivaroxaban | 36.9% | 36.9% | 36.9% | 36.9% | 26.0% | 36.9% | 36.9% |
| **Average costs/day** |  |  |  |  |  |  |  |
| VKA | 0.32 € | 0.33 € | 0.12 € | 0.12 € | 0.05 € | 0.03 € | 0.04 € |
| DOAC | 2.88 € | 3.39 € | 2.49 € | 3.43 € | 2.76 € | 3.51 € | 2.68 € |
| Contractual rebate | 10% |  |  |  |  |  |  |
| **Weighted average** | **2.14 €** | **2.51 €** | **1.80 €** | **2.47 €** | **1.87 €** | **2.50 €** | **1.91 €** |

**Table 3: Ischemic strokes, DRG data**

|  | **Germany** | **Switzerland** | **Greece** | **Netherlands** | **Poland** | **Serbia** | **UK (NHS England)** |
| --- | --- | --- | --- | --- | --- | --- | --- |
| **Publication Year** | G-DRG 2020 [10] | SwissDRG 2019 [11] | KEN DRG 2011 [12] | DBC 2018/ 2019 [15] | JGP 2016 [14] | DSG [13] | HRG 2018/ 2019 [16,30] |
| **Origin** | Australia | Australia/G-DRG | Australia |  |  | Australia |  |
| **Base rate / points** | 3530 € | 9300 CHF | 1009 € (2011) |  | 52 zl/pkt | 400 €/unit (assumption *) |  |
| **Mild complexity** | B70F 0.795 upLim 16d | B70G 0.932 upLim 15d | N30A 0.43 420 € | 15A813 (<=5d) 2,494.43 € | A50 37 pkt upLim 27d | B70D 0.53 | AA35F 1,846 UKP |
| **Moderate complexity 1** | B70D 0.948 upLim 14d | B70F 1.191 upLim 17d | N30X 1.23  1192 € | 15A828 (<=5d)  3,785.51 € | A49 77 pkt upLim 27d (>3d) | B70C 1.34 | AA35E 2,761 UKP |
| **Moderate complexity 2** | B70E 1.188 upLim 20d | B70E 1.12 upLim 13d |  | 15D621 (6-28d)  4,915.49 € |  |  | AA35D  4,047 UKP |
| **Moderate complexity 3** | B70C 1.206 upLim 15d | B70D 1.251 upLim 14d |  | 15D618 (6-28d) 7,878.30 |  |  | AA35C 5,816 UKP |
| **Moderate complexity 4** | B70B 1.4 upLim 18d | B70C 1.444 upLim 16d |  | 15A836 (6-28d) 9,466.89 € |  |  |  |
| **Moderate complexity 5** |  |  | Ν30Μβ 1.92 1860 € | 15D615 (>28d) 19,401.44 € | A48 162 pkt upLim 36d (>7d) | B70B 2.05 | AA35B  7,849 UKP |
| **High complexity 1** |  | B70B 1.722 upLim 18d |  | 15A838 (>28d) 27,751.79 € |  |  |  |
| **High complexity 2** | B70A 1.714 upLim 22d | B70A 2.422 upLim 24d | Ν30Μα 3.52 3,408 € | 15A834 (>28d) 39,088.29 € | A51 242 pkt upLim 39d (>7d) | B70A 3.95 | AA35A 12,244 UKP |

* Assumption based on cardiology case costs [31]
Table entries: Germany, Switzerland: DRG code, base relation, upLim – length of stay, upper limit; Greece: KEN DRG codes, base relation, price; Netherlands: Dbc code, length of stay, catalogue costs; Poland: JGP code, pkt: points, upLim: length of stay, upper limit; Serbia: DSG code, coefficient (units); UK: HRG code, catalogue costs

**Table 4: Synthesis of DRG data (“Inpatient cost index”)**

|  | **Germany** | **Switzerland** | **Greece** | **Netherlands** | **Poland** | **Serbia** | **UK (NHS England)** |
| --- | --- | --- | --- | --- | --- | --- | --- |
| Base relation | 3,530 € | 9,300 € | 1,009 € |  |  | 400 € |  |
| Mild complexity (22%) | 2,679 € | 8,668 € | 1,241 € | 2,494 € | 976 € | 536 € | 2,442 € |
| Moderate complexity (72%) | 4,257 € | 11,634 € | 1,937 € | 7,878 € | 2,054 € | 820 € | 5,328 € |
| High complexity (6%) | 6,050 € | 17,670 € | 3,551 € | 19,000 € | 3,068 € | 1,580 € | 9,990 € |
|  |  |  |  |  |  |  |  |
| Artificial average: Stroke hospitalization | 4,007 € | 11,344 € |  |  |  | 726 € | 4,194 € |
| Alternative sources [18,19,14] |  |  | 2,864 € | 5,328 € | 2,050 € |  |  |
| "Price per hospitalized stroke" index | 100 | 283 | 71 | 133 | 49 | 20 | 124 |

References

1. Altiok E, Marx N: **Oral Anticoagulation.** *Deutsches Arzteblatt international* 2018, **115**(46):776–783.

2. Schwabe U, Paffrath D, Ludwig W-D, Klauber J (Eds): *Arzneiverordnungs-Report 2018.* Berlin, Heidelberg: Springer Berlin Heidelberg; 2018.

3. Schwabe U, Paffrath D, Ludwig W-D, Klauber J (Eds): *Arzneiverordnungs-Report 2019.* Berlin, Heidelberg: Springer Berlin Heidelberg; 2019.

4. Schwabe U, Paffrath D (Eds): *Arzneiverordnungs-Report 2015. Aktuelle Daten, Kosten, Trends und Kommentare.* Berlin: Springer; 2015.

5. van den Heuvel JM, Hövels AM, Büller HR, Mantel-Teeuwisse AK, Boer A de, Maitland-van der Zee AH: **NOACs replace VKA as preferred oral anticoagulant among new patients: a drug utilization study in 560 pharmacies in The Netherlands.** *Thrombosis J* 2018, **16**(1):7.

6. Potpara TS, Dan G-A, Trendafilova E, Goda A, Kusljugic Z, Manola S, Music L, Musetescu R, Badila E, Mitic G, Paparisto V, Dimitrova ES, Polovina MM, Petranov SL, Djergo H, Loncar D, Bijedic A, Brusich S, Lip GYH: **Stroke prevention in atrial fibrillation and 'real world' adherence to guidelines in the Balkan Region: The BALKAN-AF Survey.** *Scientific reports* 2016, **6**(1):20432.

7. Boriani G, Proietti M, Laroche C, Fauchier L, Marin F, Nabauer M, Potpara T, Dan G-A, Kalarus Z, Diemberger I, Tavazzi L, Maggioni AP, Lip GYH: **Contemporary stroke prevention strategies in 11 096 European patients with atrial fibrillation: a report from the EURObservational Research Programme on Atrial Fibrillation (EORP-AF) Long-Term General Registry.** *Europace* 2018, **20**(5):747–757.

8. Birkemeyer R, Müller A, Wahler S, Schulenburg J-M von der: **A cost-effectiveness analysis model of Preventicus atrial fibrillation screening from the point of view of statutory health insurance in Germany.** *Health Econ Rev* 2020, **10**(1):16.

9. Ibáñez L, Sabaté M, Vidal X, Ballarin E, Rottenkolber M, Schmiedl S, Heeke A, Huerta C, Martin Merino E, Montero D, Leon-Muñoz LM, Gasse C, Moore N, Droz C, Lassalle R, Aakjaer M, Andersen M, Bruin ML de, Groenwold R, van den Ham HA, Souverein P, Klungel O, Gardarsdottir H: **Incidence of direct oral anticoagulant use in patients with nonvalvular atrial fibrillation and characteristics of users in 6 European countries (2008-2015): A cross-national drug utilization study.** *British journal of clinical pharmacology* 2019, **85**(11):2524–2539.

10. INEK Institut für Entgeltwesen im Krankenhaus: **Fallpauschalen-Katalog 2020**. [https://www.g-drg.de/aG-DRG-System_2020/Fallpauschalen-Katalog/Fallpauschalen-Katalog_2020]. [Last accessed 01 Sep 2021].

11. SwissDRG AG: *Fallpauschalen-Katalog. Planungsversion 2019/2020*; 2019.

12. Greek Republic, Ministry of Health and Social Change, Office of General Grammate: *Hospital prices K.E.N. (Κλειστά Ενοποιημένα Νοσήλια (Κ.Ε.Ν.))*; 17 June, 2011.

13. RFZO - National Health Insurance Fund: *Osnovne informacije u vezi sa Dijagnostički srodnim grupama*.

14. Narodowy Fundusz Zdrowia NFZ: *Ischemic Stroke (Udar niedokrwienny mózgu)*; November 2019.

15. Nederlandsse Zorgautoriteit: *Dbc-zorgproducten. Dbc-pakket 2020 RZ20a*.

16. NHS National Health Service: **2017/18 and 2018/19 National Tariff Payment System**. **Annex A: national tariff workbook**. [https://improvement.nhs.uk/resources/national-tariff-policy-proposals-1718-and-1819/].

17. INEK Institut für Entgeltwesen im Krankenhaus: **G-DRG-Browser 2018_2019**. [https://www.g-drg.de/Datenbrowser_und_Begleitforschung/Datenveroeffentlichung_gem._21_KHEntgG/G-DRG-Browser_2018_20192]. [Last accessed 01 Sep 2020].

18. Kritikou P, Spengos K, Zakopoulos N, Tountas Y, Yfantopoulos J, Vemmos K: **Resource utilization and costs for treatment of stroke patients in an acute stroke unit in Greece.** *Clinical neurology and neurosurgery* 2016, **142**:8–14.

19. Buisman LR, Tan SS, Nederkoorn PJ, Koudstaal PJ, Redekop WK: **Hospital costs of ischemic stroke and TIA in the Netherlands.** *Neurology* 2015, **84**(22):2208–2215.

20. MEDA Pharma GmbH & Co.KG: **Marcumar**. **Fachinformation**. [https://www.marcumar.de/fileadmin/media/Fachpublikum/Fachinformation_Marcumar.pdf]. [Last accessed June 20th, 2020].

21. Bayer AG: **Xarelto®Dosing Guide**. [https://www.xarelto.com/sites/g/files/vrxlpx171/files/2020-01/dosing-guide-xarelto-effective-protection-indications-worldwide-09-2018.pdf]. [Last accessed June 20th, 2020].

22. Kassenärztlicher Bundesverband: *Direkte orale Antikoagulanzien: Tiefe Venenthrombosen und Lungenembolien*.

23. **DocMorris Apotheke**. [https://www.docmorris.de]. [Last accessed June 20th, 2020].

24. **Adler Apotheke Winterthur**. [https://www.adlershop.ch/c/1229/arzneimittel]. [Last accessed June 20th, 2020].

25. **Γαληνός - Οδηγός φαρμάκων (Galinos medication guide)**. [https://www.galinos.gr/]. [Last accessed June 20th, 2020].

26. Ellinikí Dimokratía: Ministry of Health: **Δελτία Τιμών (Price sheets)**. [https://www.moh.gov.gr/articles/times-farmakwn/deltia-timwn].

27. RFZO: National Helth Insurance Fund: **Листа лекова (Листе А, А1, Б, Ц и Д) (Lista lekova)**. [https://www.rfzo.rs/index.php/osiguranalica/lekovi-info/lekovi-actual]. [Last accessed June 20th, 2020].

28. **Medycyna praktyczna dla pacjentów**. **Baza leków**. [https://www.mp.pl/pacjent/leki/]. [Last accessed June 20th, 2020].

29. Zorginstituut Nederland: **Farmacotherapeutisch Kompas: Kostenoverzicht direct werkende orale anticoagulantia**.

30. NHS National Health Service: **Hospital Accident and Emergency Activity - 2018-19**. [https://digital.nhs.uk/data-and-information/publications/statistical/hospital-accident--emergency-activity/2018-19].

31. Lakić D, Petrova G, Bogavac-Stanojević N, Jelić-Ivanović Z, Kos M: **The Cost-Effectiveness of Hypertension Pharmacotherapy in Serbia: A Markov Model.** *Biotechnology & Biotechnological Equipment* 2012, **26**(3):3066–3072.
